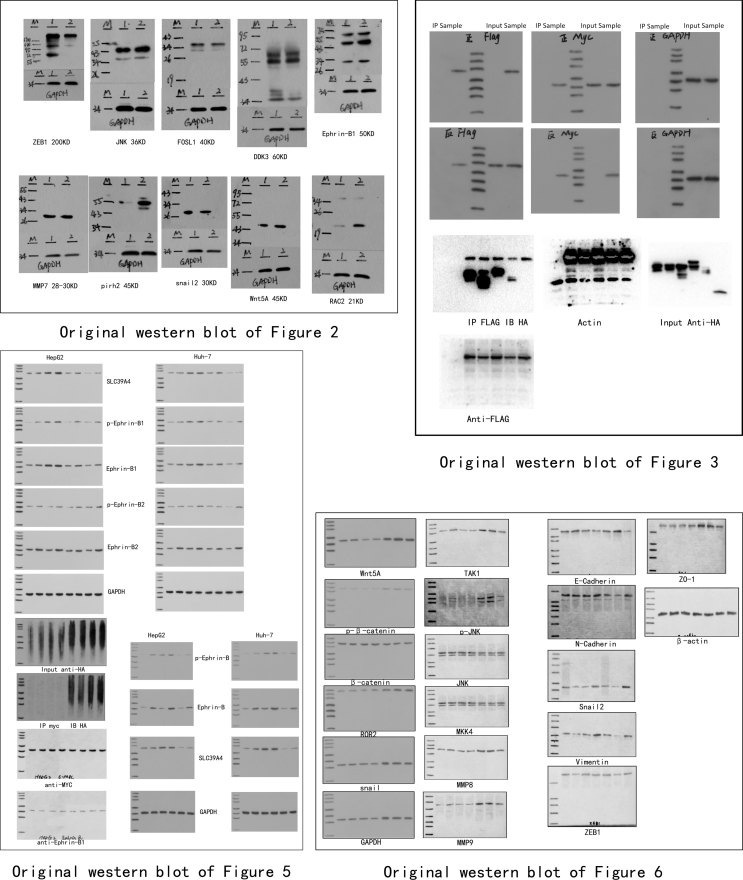# ZIP4 inhibits Ephrin-B1 ubiquitination, activating Wnt5A/JNK/ZEB1 to promote liver cancer metastasis

**DOI:** 10.1016/j.gendis.2024.101312

**Published:** 2024-04-24

**Authors:** Haijun Guo, Renyi Su, Xinfeng Lu, Hui Zhang, Xuyong Wei, Xiao Xu

**Affiliations:** a Department of Hepatobiliary and Pancreatic Surgery, Affiliated Hangzhou First People's Hospital, Zhejiang University School of Medicine, Hangzhou, Zhejiang 310006, China; b Zhejiang University School of Medicine, Hangzhou, Zhejiang 310058, China; c The Fourth School of Clinical Medicine, Zhejiang Chinese Medicine University, Hangzhou, Zhejiang 310053, China; d School of Clinical Medicine, Hangzhou Medical College, Hangzhou, Zhejiang 310014, China

Hepatocellular carcinoma (HCC), a highly malignant tumor, faces a major challenge with its high post-hepatectomy recurrence impacting patient survival. Mechanisms and preventive strategies remain to be resolved. Zrt-/Irt-like protein 4 (ZIP4, also called SLC39A4/solute carrier family 39 member 4) belongs to the zinc transporter ZIP superfamily of which the members play an important role in maintaining zinc steady state, and its high expression in various tumors is associated with the prognosis of cancer patients.^1^ Recently, it has been reported that ZIP4 promoted the development of pancreatic cancer through the zinc finger E-box binding homeobox 1 (ZEB1)/Integrin α3β1/equilibrative nucleoside transporter (ENT1) ^1^^,^^2^ and zona occludens 1 (ZO-1)/claudin-1/ZEB1 pathways.^3^ In nasopharyngeal carcinoma, ZIP4 induces epithelial–mesenchymal transition (EMT) through the phosphatidylinositol 3-kinase (PI3K)/protein kinase B (AKT) signaling pathway and promotes migration and invasion.^4^ In our previous study, ZIP4 was found to be highly expressed in HCC and promoted HCC invasion and metastasis.^5^ But so far, the direct regulation mechanism for ZIP4 promotes liver cancer is still unclear. In this study, we found that ZIP4 binds to Ephrin-B1 to inhibit the ubiquitination of Ephrin-B1, regulating the Wnt family member 5A (Wnt5A)/Jun N-terminal kinase (JNK)/ZEB1 signaling pathway and promoting EMT, thereby inducing invasion and metastasis of liver cancer cells.

We established stable cell lines with ZIP4 overexpression and knockdown lentivirus, the interference efficiency of ZIP4 reached 66.1% and ZIP4 expression increased by 50 times after overexpression (Fig. S1A). Using cDNA gene microarrays, we identified 1677 DEGs (differentially expressed genes) (Fig. S1B) in the ZIP4 interference group (805 up-regulated and 872 down-regulated genes) (Fig. 1A). Metascape enrichment analysis revealed that ZIP4 was significantly associated with tyrosine kinase receptor signaling, cell migration regulation, and EMT pathways (Fig. S1D, E). Cluster analysis was performed on 40 DEGs (fold change >2.5) (Fig. S1C), and their expression was verified by Western blot and reverse-transcription PCR (Fig. S1G, H, 2). The results showed that the levels of Ephrin-B1, Wnt5A, and JNK were lower after ZIP4 knockout. Through the Uniprot database, we found 163 proteins were predicted to interact with ZIP4. These proteins were intersected with DEGs, and 23 common proteins were found (Fig. S1F). We then performed a cluster analysis of 23 common differentially expressed genes (Fig. 1B). Ephrin-B1 is a member of the tyrosine kinase receptor family, and with the enrichment analysis and reported gene functions, we hypothesized that Ephrin-B1 was a candidate to interact with ZIP4.

**Figure 1:**
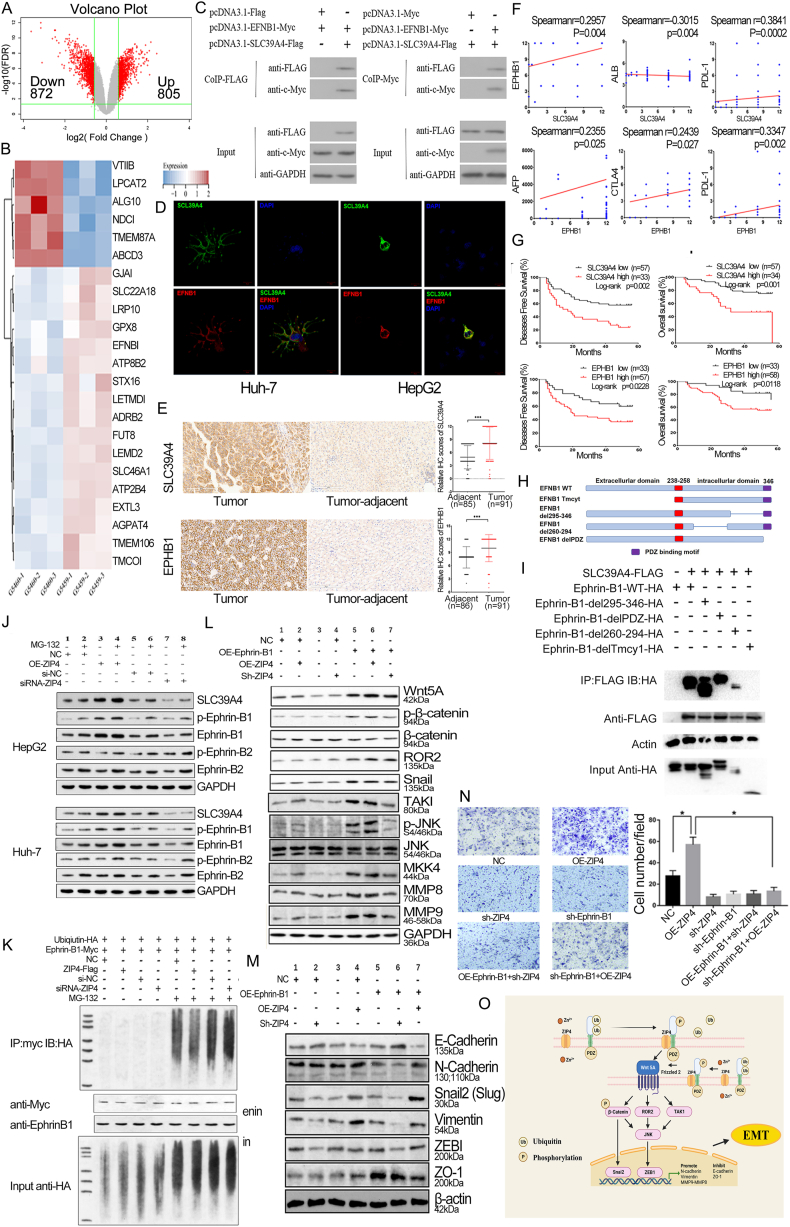
ZIP4 inhibits ubiquitination of Ephrin-B1 to activate Wnt5A/JNK/ZEB1 and induce EMT of hepatocellular carcinoma. **(A)** Volcano map of differentially expressed genes (DEGs) after transfection with interference lentivirus (sh-ZIP4). The red dots are the significantly DEGs screened by fold change >1.5 and false discovery rate <0.05. A total of 805 up-regulated genes and 872 down-regulated genes are on the right and left of the figure, respectively. **(B)** Cluster analysis was performed on 23 common genes. **(C)** Co-immunoprecipitation showed that ZIP4 bound to Ephrin-B1. **(D)** In immunofluorescence co-localization, ZIP4 is shown in green, Ephrin-B1 in red, and DAPI in blue (the nucleus). ZIP4 binds to Ephrin-B1 in the cell membrane and emits yellow fluorescence in Huh-7 and HepG2 cell lines. **(E)** Images of ZIP4 (SLC39A4) and Ephrin-B1 (EPHB1) expression by immunohistochemistry analysis in tissue microarrays. The expression of ZIP4 and Ephrin-B1 in HCC tissues was significantly higher than those in adjacent tissues (*P* < 0.005). **(F)** The expression of ZIP4 was linearly correlated with Ephrin-B1, albumin (ALB), and programmed cell death ligand 1 (PD-L1). The expression of Ephrin-B1 was linearly correlated with alpha-fetoprotein (AFP), cytotoxic T lymphocyte antigen-4 (CTLA4), and PD-L1. **(G)** The overall survival rate and disease-free survival rate of liver cancer patients with high expression of ZIP4 were lower. The overall survival rate and disease-free survival rate of liver cancer patients with high expression of Ephrin-B1 were lower. **(H)** WT-Eprhin-B1 (wild type) or deletion mutant Eprhin-B1 with amino acids 260–294 or 295–346 or PDZ-binding motif in the intracellular domain or extracellular domain (Tmcy1). **(I)** HEK293T cells were co-transfected with Flag-tagged ZIP4 (SLC39A4) along with WT or deletion mutants of Ephrin-B1 as indicated. Cell lysates were immunoprecipitated with HA antibody. Immunoprecipitates and total lysates were immunoblotted with HA or Flag antibodies. **(J)** Overexpression of ZIP4 enhanced the expression of Ephrin-B1 and phosphorylated Ephrin-B1 after the addition of the ubiquitinase inhibitor. Ephrin-B2 and phosphorylated Ephrin-B2 expression showed no significant change after ZIP4 overexpression and interference. **(K)** Overexpression of ZIP4 reduced the level of ubiquitin in immune complexes that co-immunoprecipitated Ephrin-B1, and inhibition of ZIP4 increased the level of ubiquitin in immune complexes that co-immunoprecipitated Ephrin-B1. **(L)** The protein expression of ZIP4 and Ephrin-B1 downstream pathways was verified. Western blot showed that Wnt5A, Snail, ROR2, MKK4, MMP8, MMP9, TAK1, and p-JNK expression were also increased and decreased after ZIP4 overexpression or suppression. **(M)** ZIP4 and Ephrin-B1 regulated the key molecular markers of EMT (β-catenin, vimentin, E-cadherin, N-cadherin, Snail2, ZEB1). **(N)** Invasion recovery assay confirmed that Ephrin-B1 was a key downstream molecule of ZIP4. **(O)** Schemata of downstream pathways that promote EMT in liver cancer after ZIP4 binding to Ephrin-B1. EMT, epithelial–mesenchymal transition; JNK, Jun N-terminal kinase; MKK4, mitogen-activated protein kinase kinase 4; MMP8/9, matrix metallopeptidase 8/9; ROR2, receptor tyrosine kinase-like orphan receptor 2; TAK1, transforming growth factor β-activated kinase 1; Wnt5A, Wnt family member 5A; ZEB1, zinc finger E-box binding homeobox 1.

Immunoprecipitation results showed that Ephrin-B1 interacted with ZIP4 (Fig. 1C; Fig. S2). By immunofluorescence, we also found that Ephrin-B1 co-located with ZIP4 (Fig. 1D). Then we constructed a series of deletion mutants of Ephrin-B1, and the data of co-immunoprecipitation showed that deletion of the extracellular domain (Tmcy1) of Ephrin-B1 inhibited the binding of ZIP4 to Ephrin-B1 (Fig. 1H, I; Fig. S2). These data suggest that ZIP4 binds to Tmcy1, the extracellular domain of Eprhin-B1.

Whether there is a relationship between the expression of ZIP4 and Ephrin-B1 in HCC patients? ZIP4 and Ephrin-B1 levels were highly expressed in HCC tissues (Fig. 1E). The expression of ZIP4 was significantly correlated with Ephrin-B1, age, programmed cell death ligand 1 (PD-L1) (Table S1, *P* < 0.05). The expression of Ephrin-B1 was significantly correlated with alpha-fetoprotein, PD-L1, and cytotoxic T lymphocyte antigen-4 (Table S2, *P* < 0.05). Linear correlation analysis showed that ZIP4 expression was highly correlated with Ephrin-B1, albumin, and PD-L1. Meanwhile, Ephrin-B1 was highly linearly correlated with the levels of alpha-fetoprotein, PD-L1, and cytotoxic T lymphocyte antigen-4 (Fig. 1I). The Kaplan–Meier survival curve showed overall and tumor-free survival rates were lower in patients with higher ZIP4 and/or Ephrin-B1 expression (Fig. 1F). Multivariate analyses showed that ZIP4 and Ephrin-B1 expression were independent prognostic factors for overall survival (hazard ratio = 2.778, *P* = 0.004; hazard ratio = 2.441, *P* = 0.045) (Table S3). ZIP4 expression was an independent prognostic factor for disease-free survival (hazard ratio = 2.229, *P* = 0.006) (Table S4).

The effect of ZIP4 on ubiquitination was investigated by adding ubiquitination enzyme inhibitors (MG-132) when ZIP4 was interfered with and overexpressed. Ephrin-B1 and phosphorylated Ephrin-B1 (p-Ephrin-B1) expression increased significantly after ZIP4 overexpression. Their expression also significantly increased when the ubiquitinase inhibitor MG-132 was added. Their expression decreased after interfering with ZIP4. Their expression increased after the inhibition of ZIP4 and the addition of MG-132 (Fig. 1J; Fig. S2). The addition of MG132 reversed the effect of ZIP4 inhibition on Ephrin-B1 and p-Ephrin-B1. These results suggest that the ubiquitinase inhibitor and ZIP4 exert the same effect on Ephrin-B1 expression. Moreover, when comparing the regulatory effect of ZIP4 overexpression or inhibition in the presence of MG132, we found that ZIP4 overexpression significantly promoted the expression of Ephrin-B1 and p-Ephrin-B1, but ZIP4 inhibition did not affect their expression. Therefore, we hypothesized that ZIP4 did not directly regulate the expression of Ephrin-B1 and p-Ephrin-B1, but inhibited their ubiquitination and degradation. Then we used HA-tagged Ubiquitin and MyC-tagged Ephrin-B1 to transfect the corresponding plasmids into HEK293T cells. Co-immunoprecipitation was performed using antibodies to HA and MYC. Ubiquitin expression was measured with antibodies against HA in Myc-tagged Ephrin-B1 immune complexes. The results showed that the ubiquitin content was significantly increased after the use of the ubiquitin enzyme inhibitor. Overexpression of ZIP4 reduced the expression of ubiquitin-binding to Ephrin-B1, but inhibition of ZIP4 increased the expression of ubiquitin-binding to Ephrin-B1 (Fig. 1K; Fig. S2). The above results directly confirmed that the binding of ZIP4 to Ephrin-B1 inhibited its ubiquitination degradation.

To verify that ZIP4 regulated the invasion function of HCC cells via Ephrin-B1, we performed a reversion assay. Consistent with the results of our earlier study, the number of invasive cells was significantly increased after ZIP4 overexpression. After interfering with ZIP4, the invasion of HCC cells was significantly reduced. After interfering with Ephrin-B1, the number of invasive cells was also significantly reduced. However, overexpression of ZIP4 and interference of Ephrin-B1 expression resulted in a significantly lower number of HCC invasion cells than the ZIP4 overexpression group and the control group (Fig. 1N). These results indicate that Ephrin-B1 is a key molecule downstream of ZIP4.

The expression changes of downstream EMT pathway proteins were explored by overexpression and inhibition of ZIP4 and overexpression of Ephrin-B1 to rescue the effect of ZIP4 inhibition. Results from Western blot showed that Wnt5A, Snail, receptor tyrosine kinase-like orphan receptor 2 (ROR2), Snail2, mitogen-activated protein kinase kinase 4 (MKK4), matrix metallopeptidase (MMP)8, MMP9, transforming growth factor β-activated kinase 1, p-JNK, ZEB1, and vimentin expression was also increased and decreased after ZIP4 overexpression or suppression. The expression of E-cadherin and zona occludens 1 was decreased upon ZIP4 overexpression and increased upon ZIP4 inhibition. Knockdown of ZIP4 with overexpression of Ephrin-B1 up-regulated Wnt5A, p-β-catenin, Snail, ROR2, MKK4, MMP8, MMP9, ZEB1, p-JNK, and Snail2 expression (Fig. 1L, M; Fig. S2). These results suggest that Ephrin-B1 is able to rescue the effects of ZIP4 knockdown on downstream pathways. ZIP4 regulates the expression of downstream proteins through the regulation of Ephrin-B1. ZIP4 regulated the expression of EMT markers (β-catenin, E-cadherin, N-cadherin, Snail, Snail2, ZEB1, and vimentin), indicating that ZIP4 regulation induced EMT in liver cancer. Combined with previous reports on the Wnt pathway, EMT pathway, and our results, we mapped the ZIP4 signaling pathway (Fig. 1O).

In summary, we report for the first time that ZIP4 interacts with Ephrin-B1 and regulates the ubiquitination of Ephrin-B1 to affect the downstream Wnt5A/JNK/ZEB1 signaling pathway, thereby promoting EMT and invasion and metastasis of HCC cells. *In vivo*, it was found that ZIP4 and Ephrin-B1 expression were linearly correlated, and ZIP4 and Ephrin-B1 were independent risk factors for overall survival after liver cancer surgery. ZIP4 and Ephrin-B1 can be used as potential molecular targets for the treatment of liver cancer.

## Ethics declaration

The study was approved by the Ethics Committee of Hangzhou First People's Hospital (No. 2022-010-01). Written informed consent was obtained from all participants in this study.

## Author contributions

H.J.G., R.Y.S., and X.X. designed this study. R.Y.S. and X.F.L. performed the experiments. H.Z. and X.F.L. acquired data. H.J.G. and X.Y.W. analyzed and interpreted the data. H.J.G. wrote the paper. H.J.G., X.Y.W., and X.X. critically reviewed the manuscript. All authors read and approved the final manuscript.

## Conflict of interests

The authors declared no conflict of interests.

## Funding

This work was supported by 10.13039/501100004731Zhejiang Provincial Natural Science Foundation of China (No. LQ20H160029), The Construction Fund of Key Medical Disciplines of Hangzhou, Zhejiang, China (No. OO20200093), The Major Research Plan of the National Natural Science Foundation of China (No.92159202), The Key Program, National Natural Science Foundation of China (No. 81930016), and The 10.13039/100022963Key Research & Development Plan of Zhejiang Province, China (No. 2019C03050).

## Figures and Tables

**figs1:**
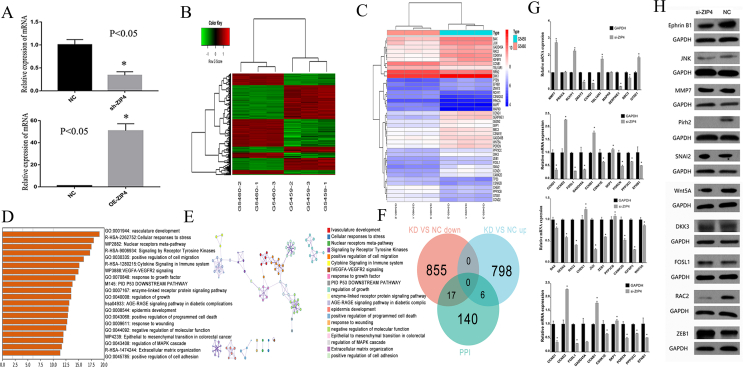


**figs2:**