# Supplementary material for: ZIP4 inhibits Ephrin-B1 ubiquitination, activating Wnt5A/JNK/ZEB1 to promote liver cancer metastasis
Source: Genes Dis. 2024 Apr 24;11(6):101312. doi: 10.1016/j.gendis.2024.101312 (PMC11260364; doi:10.1016/j.gendis.2024.101312)
Supplement: Multimedia component 1 [file mmc1.docx]

**Supplementary Materials and methods**

**Abbreviations:**

HCC, Hepatocellular Carcinoma; EMT, Epithelial-mesenchymal transition; RT-PCR, reverse transcription-polymerase chain reaction; ZIPS, ZRT-IRF-Like Proteins; ZIP4, Zinc ion transporter 4; FDR, False Discovery Rate; DEGs, differentially expressed genes; IPA, Ingenuity Pathway Analysis; PD-L1, programmed cell death-Ligand 1; CTLA4, cytotoxic T-lymphocyte-associated protein 4; ALB, albumin; OS, Overall Survival; DFS, Disease-Free Survival; Tyr, tyrosine; TB, total bilirubin; ALT, alanine transaminase; GGT, γ-glutamyl transpeptidase; NC, negative control;

**Availability of data and materials**

The data that support our findings are available from the corresponding author upon reasonable request.

**Acknowledgments**

Thank you for the technical support from the Core Facilities of Zhejiang University School of Medicine. The authors wish to express their gratitude to all the staff of Key Laboratory of Integrated Oncology and Intelligent Medicine of Zhejiang Province.

**Consent for publication**

All authors agree to publish this paper.

**Cells cultured**

HEK293T, HCC cell line HepG2, and Huh-7 were purchased from the Cell Bank of the Chinese Academy of Sciences (Shanghai, China). The cells were cultured in DMEM medium supplemented with 10% FBS, 1% penicillin, and 1% streptomycin.

**Construction of stable transfected cells**

Lentivirus vector (LVpFU-GW-007) of ZIP4 was provided by GeneChem (Shanghai, China). The overexpression sequence was full-length ZIP4 (NM_130849). The interference sequence is: 5’-GGATGACATCACCTTCTGA-3’, 3’-TTGAGGCTACTGCCCTATA-5’. The lentivirus-expressing shZIP4 and OE-ZIP4 were used to infect HepG2 cells for construction ZIP4 stable knockdown and overexpression cells. The virus vector was transfected into the cells as a NC. Cells infected with lentivirus were screened for 2 weeks by adding screening antibiotics (1 μg/ml puromycin (Sigma, MO, USA) two days after infection.

**Transfection**

The full-length sequences of Ephrin-B1 (NM_004429) and ZIP4 (BC062625.1) were inserted into the vector pcDNA-3.1 to synthesize the overexpression vector of Ephrin-B1 (OE-Ephrin-B1) and ZIP4 (OE-ZIP4). The interfering sequences of ZIP4 and Ephrin-B1 inserted into vector pcDNA-3.1 were called sh-ZIP4 and sh-Ephrin-B1. The empty vector pcDNA-3.1 was transfected into cells as NC. Cells were transfected using the Lipofectamine 2000 (Invitrogen, CA, USA).

**RNA extraction and** **RT-PCR**

The RNA was extracted using TRIzol reagent (Invitrogen) and analyzed by Nano Drop 2000 (Thermo Fisher Scientific, MA, USA), then RNA was reverse transcribed to cDNA according to the M-MLV Reverse Transcriptase (TaKaRa, Dalian, China) product specification. SYBR Green PCR Master Mix (Applied Biosystems, CA, USA) was used to complete RT-PCR. The corresponding primer sequences are provided in Supplement Table 5. The 2-ΔΔCt method was used to calculate the relative RNA expression. Each test was run in triplicate and GAPDH was used as an internal control.

**Microarray Analysis**

Affymetrix GeneChip® Prime View Human Gene Expression Array was used to perform microarray analysis. Raw data was normalized using SST-RMA algorithm. Gene expression levels were measured using GeneSpring software (Agilent Technologies, CA, USA). For genes that were upregulated or downregulated more than 1.5-fold after ZIP4 overexpression and interference, we further analyzed the data by IPA (QIAGEN, CA, USA) online tool to predict potential upstream regulators and classical signaling pathways.

**Immunoprecipitation and Western Blot**

Total proteins were lysed using RIPA Lysis Buffer (FUDEBIO, Hangzhou, China). Equivalent amounts of protein lysate were incubated with anti-HA (Abcam, ab9110), anti-Flag (Sigma), anti-Myc (Abcam, ab9106) or anti-EphrinB1 (Abcam, ab99029) overnight at 4℃ with shaking. Then, protein-A/G-agarose (Santa Cruz Biotechnology) was used to incubate at 4℃ for 1 hour. Protein lysates was washed three times with lysis buffer. After co-immunoprecipitation and immunoblotting were separated by SDS-PAGE, samples were transferred to PVDF membranes. After incubation with primary antibody overnight and secondary antibody for 1 hour, PVDF membranes were visualized using chemiluminescence reagents. The primary antibodies are shown in Supplementary Table 6.

**Confocal immunofluorescence analysis**

Cells (5 × 10^3^) were fixed with paraformaldehyde for 30 min after 24 h of culture. This was followed by incubation with 0.1% Triton X-100 at room temperature for 5 min. The primary anti-human ZIP4 antibody (Sigma; dilution 1:200) and anti-human Ephrin-B1 antibody was used to incubate with in the dark for 24 hours at 4℃. The cells were incubated with green fluorescent and red fluorescent secondary antibody for 1 hour at room temperature. Cells were stained using 10 μg/ml DAPI and subsequently covered with a cover glass and photographed under a fluorescence microscope.

**Transwell assay**

Cells were counted at 5 × 10^4^/ml cells and cultured in the upper chambers with serum-free medium. Transwell chamber (Millipore, Billerica, USA) coated with Matrigel were used to evaluate cell invasion. After incubation for 48 h with culture medium containing 10% FBS in the lower chamber, cells at the bottom of the upper chamber were wiped off with a cotton swab. Cells on the reverse side of the bottom of the upper chamber were fixed with 4% paraformaldehyde and stained with 0.1% crystal violet. Five fields were randomly selected and stained cells were photographed under a light microscope.

**Patients and tissues specimen**

A total of 92 patients who were diagnosed with HCC from 2018 to 2020 in the Hangzhou First People’s Hospital, affiliated to Zhejiang University of Medicine were enrolled. None of the patients received chemotherapy or radiotherapy or immunotherapy before the surgery. Tumor and adjacent tissues were collected during surgery. The enrolled patients were followed up by telephone. All patients provided written informed consent for enrollment. The ethics approval committee of Hangzhou First People's Hospital approved this study.

**Tissue microarrays analysis**

Anti-human ZIP4 antibody and Anti-human Ephrin-B1 antibody was used for Immunohistochemistry according to a method described previously [29]. Two pathologists independently scored the intensity of immunostaining. Stained sections were visualized under a microscope 200× magnifications. At least 10 randomly selected fields were selected for each section. The staining intensity score was determined by two factors: the staining intensity and the area of the stained area. The scoring method was the same as described previously ^29^.

**Statistical analysis**

Statistical analysis used SPSS 19.0 software (IBM, IL, USA). The results are shown as means ± standard deviations. Statistical comparison was analyzed using Student’s t tests or chi-square test or one-way ANOVA. Survival analysis patients with HCC was evaluated based on ZIP4 and Ephrin-B1 expression using Kaplan-Meier analysis. The correlation of ZIP4 and Ephrin-B1 levels in HCC tissues was analyzed by linear regression analysis using sing GraphPad Prism version 7.0 (GraphPad software, San Diego, CA, USA). A value of *P* <0.05 was considered statistically significant.

**Supplementary Table 1**

**Supplementary Table1** Correlation between **SLC39A4** expression and clinicopathological characteristics

|  | variables | SLC39A4 expression | | total | χ2 | p value |
| --- | --- | --- | --- | --- | --- | --- |
|  |  | High | Low |  |  |  |
| Age (year) |  |  |  |  | 6.048 | 0.014 |
|  | <50 | 17 | 19 | 36 |  |  |
|  | ＞=50 | 40 | 15 | 55 |  |  |
| Sex |  |  |  |  | 0.024 | 0.878 |
|  | Female | 6 | 4 | 10 |  |  |
|  | male | 50 | 30 | 80 |  |  |
| Grade |  |  |  |  | 3.760 | 0.052 |
|  | I-II | 37 | 15 | 53 |  |  |
|  | III | 20 | 19 | 38 |  |  |
| T stage |  |  |  |  | 0.567 | 0.452 |
|  | T1 | 38 | 20 | 58 |  |  |
|  | T2-T3 | 19 | 14 | 33 |  |  |
| TNM stage |  |  |  |  | 0.567 | 0.452 |
|  | I | 38 | 20 | 58 |  |  |
|  | II-III | 19 | 14 | 33 |  |  |
| Tumor capsule |  |  |  |  | 3.722 | 0.054 |
|  | complete | 31 | 11 | 42 |  |  |
|  | incomplete | 26 | 22 | 48 |  |  |
| cirrhosis |  |  |  |  | 0.898 | 0.343 |
|  | negative | 7 | 2 | 9 |  |  |
|  | positive | 50 | 31 | 81 |  |  |
| HBsAg |  |  |  |  | 0.268 | 0.604 |
|  | negative | 13 | 6 | 19 |  |  |
|  | positive | 44 | 27 | 71 |  |  |
| HBcAb |  |  |  |  | 1.193 | 0.275 |
|  | negative | 3 | 4 | 7 |  |  |
|  | positive | 51 | 29 | 80 |  |  |
| AntiHCV |  |  |  |  | # | 1.000 |
|  | negative | 53 | 33 | 86 |  |  |
|  | positive | 1 | 0 | 1 |  |  |
| TB |  |  |  |  | 0.157 | 0.692 |
|  | negative | 41 | 25 | 66 |  |  |
|  | positive | 16 | 8 | 24 |  |  |
| ALT |  |  |  |  | 0.965 | 0.326 |
|  | negative | 32 | 22 | 54 |  |  |
|  | positive | 25 | 11 | 36 |  |  |
| ALB |  |  |  |  | 0.000 | 1.000 |
|  | negative | 55 | 32 | 87 |  |  |
|  | positive | 2 | 1 | 3 |  |  |
| AFP |  |  |  |  | 0.329 | 0.566 |
|  | negative | 26 | 13 | 39 |  |  |
|  | positive | 31 | 20 | 51 |  |  |
| GGT |  |  |  |  | 0.246 | 0.620 |
|  | negative | 28 | 18 | 46 |  |  |
|  | positive | 29 | 15 | 44 |  |  |
| PDL-1 |  |  |  |  | 10.505 | 0.001 |
|  | negative | 24 | 4 | 28 |  |  |
|  | positive | 27 | 28 | 55 |  |  |
| CTLA4 |  |  |  |  | 0.939 | 0.332 |
|  | negative | 23 | 10 | 33 |  |  |
|  | positive | 29 | 20 | 49 |  |  |
| Ephrin-B1 |  |  |  |  |  |  |
|  | High | 31 | 27 | 58 | 5.771 | 0.016 |
|  | Low | 26 | 8 | 34 |  |  |

* Statistically significant(p<0.05)

**Supplementary Table 2**

**Supplementary Table 2** Correlation between **Ephrin-B1** expression and clinicopathological characteristics

|  | variables | **Ephrin-B1** expression | | total | χ2 | p value |
| --- | --- | --- | --- | --- | --- | --- |
|  |  | Low | High |  |  |  |
| Age (year) |  |  |  |  | 0.544 | 0.461 |
|  | <50 | 12 | 25 | 37 |  |  |
|  | ＞=50 | 22 | 33 | 55 |  |  |
| Sex |  |  |  |  | 0.280 | 0.597 |
|  | Female | 5 | 5 | 10 |  |  |
|  | male | 29 | 52 | 80 |  |  |
| Grade |  |  |  |  | 1.470 | 0.225 |
|  | I-II | 22 | 30 | 52 |  |  |
|  | III | 12 | 28 | 40 |  |  |
| T stage |  |  |  |  | 0.008 | 0.930 |
|  | T1 | 22 | 37 | 59 |  |  |
|  | T2-T3 | 12 | 21 | 33 |  |  |
| TNM stage |  |  |  |  | 0.008 | 0.930 |
|  | I | 22 | 37 | 59 |  |  |
|  | II-III | 12 | 21 | 33 |  |  |
| Tumor capsule |  |  |  |  | 0.018 | 0.894 |
|  | complete | 16 | 26 | 42 |  |  |
|  | incomplete | 18 | 31 | 49 |  |  |
| cirrhosis |  |  |  |  | 0.681 | 0.409 |
|  | negative | 5 | 4 | 9 |  |  |
|  | positive | 29 | 53 | 82 |  |  |
| HBsAg |  |  |  |  | 0.027 | 0.311 |
|  | negative | 9 | 10 | 19 |  |  |
|  | positive | 25 | 47 | 72 |  |  |
| HBcAb |  |  |  |  | 0.027 | 0.869 |
|  | negative | 2 | 5 | 7 |  |  |
|  | positive | 32 | 49 | 81 |  |  |
| AntiHCV |  |  |  |  | 0.984 | 0.321 |
|  | negative | 34 | 53 | 87 |  |  |
|  | positive | 0 | 1 | 1 |  |  |
| TB |  |  |  |  | 0.226 | 0.634 |
|  | negative | 26 | 41 | 67 |  |  |
|  | positive | 8 | 16 | 24 |  |  |
| ALT |  |  |  |  | 0.269 | 0.604 |
|  | negative | 19 | 35 | 54 |  |  |
|  | positive | 15 | 22 | 37 |  |  |
| ALB |  |  |  |  | 0.000 | 1.000 |
|  | negative | 33 | 55 | 88 |  |  |
|  | positive | 1 | 2 | 3 |  |  |
| AFP |  |  |  |  | 5.650 | 0.017 |
|  | negative | 20 | 19 | 39 |  |  |
|  | positive | 14 | 38 | 52 |  |  |
| GGT |  |  |  |  | 2.384 | 0.123 |
|  | negative | 14 | 33 | 47 |  |  |
|  | positive | 20 | 24 | 44 |  |  |
| PDL-1 |  |  |  |  | 4.266 | 0.039 |
|  | negative | 15 | 13 | 28 |  |  |
|  | positive | 17 | 39 | 56 |  |  |
| CTLA4 |  |  |  |  | 4.421 | 0.035 |
|  | negative | 16 | 17 | 33 |  |  |
|  | positive | 13 | 37 | 50 |  |  |

* Statistically significant(p<0.05)

**Supplementary Table 3**

**Supplementary Table 3** Univariate and multivariate analyses of the factors correlated with Overall survival of Liver carcinoma patients

| Variables | Univariate analysis | | |  | Multivariate analysis | | |  |
| --- | --- | --- | --- | --- | --- | --- | --- | --- |
|  | HR | 95%CI | p value |  | HR | 95%CI | p value |  |
| ZIP4 | 3.125 | 1.557-6.271 | 0.001 |  | 2.778 | 1.373-5.622 | 0.004 |  |
| Ephrin-B1 | 2.944 | 1.249-6.940 | 0.014 |  | 2.441 | 1.020-5.839 | 0.045 |  |
| Sex | 1.972 | 0.472-8.241 | 0.352 |  |  |  |  |  |
| Age(＞=50) | 1.175 | 0.578-2.389 | 0.656 |  |  |  |  |  |
| Grade | 1.288 | 0.735-2.257 | 0.376 |  |  |  |  |  |
| T stage | 1.956 | 0.987-3.875 | 0.055 |  |  |  |  |  |
| TNM stage | 1.956 | 0.987-3.875 | 0.055 |  |  |  |  |  |
| tumor capsule | 1.995 | 0.960-4.145 | 0.064 |  |  |  |  |  |
| cirrhosis | 1.776 | 0.424-7.434 | 0.432 |  |  |  |  |  |
| HBsAg | 0.975 | 0.418-2.276 | 0.953 |  |  |  |  |  |
| HBcAb | 0.585 | 0.203-1.682 | 0.320 |  |  |  |  |  |
| TB | 1.075 | 0.497-2.323 | 0.855 |  |  |  |  |  |
| ALT | 1.375 | 0.686-2.756 | 0.369 |  |  |  |  |  |
| ALB | 0.736 | 0.098-5.509 | 0.765 |  |  |  |  |  |
| AFP | 1.590 | 0.766-3.302 | 0.213 |  |  |  |  |  |
| GGT | 1.756 | 0.866-3.559 | 0.119 |  |  |  |  |  |
| PDL-1 | 1.051 | 0.503-2.194 | 0.895 |  |  |  |  |  |
| CTLA4 | 0.512 | 0.208-1.265 | 0.147 |  |  |  |  |  |

* Statistically significant(p<0.05)

**Supplementary Table 4**

**Supplementary Table 4** Univariate and multivariate analyses of the factors correlated with Disease-Free Survival of Liver carcinoma patients

| Variables | Univariate analysis | | |  | Multivariate analysis | | |  |
| --- | --- | --- | --- | --- | --- | --- | --- | --- |
|  | HR | 95%CI | p value |  | HR | 95%CI | p value |  |
| ZIP4 | 2.448 | 1.394-4.298 | 0.002 |  | 2.229 | 1.259-3.946 | 0.006 |  |
| Ephrin-B1 | 2.854 | 1.122-4.002 | 0.021 |  | 1.780 | 0.934-3.393 | 0.080 |  |
| Sex | 1.289 | 0.463-3.588 | 0.627 |  |  |  |  |  |
| Age(＞=50) | 1.257 | 0.703-2.248 | 0.440 |  |  |  |  |  |
| Grade | 2.089 | 1.187-3.677 | 0.376 |  |  |  |  |  |
| T stage | 1.656 | 0.942-2.911 | 0.080 |  |  |  |  |  |
| TNM stage | 1.656 | .942-2.911 | 0.080 |  |  |  |  |  |
| tumor capsule | 1.391 | 0.786-2.461 | 0.257 |  |  |  |  |  |
| cirrhosis | 1.786 | 0.555-5.746 | 0.331 |  |  |  |  |  |
| HBsAg | 1.077 | 0.537-2.158 | 0.835 |  |  |  |  |  |
| HBcAb | 0.736 | 0.291-1.857 | 0.516 |  |  |  |  |  |
| TB | 0.798 | 0.416-1.532 | 0.498 |  |  |  |  |  |
| ALT | 1.154 | 0.657-2.026 | 0.619 |  |  |  |  |  |
| ALB | 0.472 | 0.065-3.420 | 0.457 |  |  |  |  |  |
| AFP | 1.381 | 0.777-2.454 | 0.271 |  |  |  |  |  |
| GGT | 1.577 | 0.897-2.771 | 0.114 |  |  |  |  |  |
| PDL-1 | 1.234 | 0.685-2.224 | 0.483 |  |  |  |  |  |
| CTLA4 | 0.527 | 0.259-1.073 | 0.077 |  |  |  |  |  |

* Statistically significant(p<0.05)

**Supplement Table 5**

**Supplement Table 5** The corresponding primer sequences of RT-PCR

| **Target gene** | **Sequence of upstream primers** | **Sequence of downstream primers** |
| --- | --- | --- |
| MMP7 | GGGGACTCCTACCCATTTG | TCCAGCGTTCATCCTCATC |
| PRKCA | GTAGTGTCAGGGATTCTTCG | CTGTTGGATGGCTGAGTG |
| RCHY1 | TAGAGGCTACAGATGTCCATTA | GCAGAGAATATCCACAGTCAT |
| ZMAT3 | GCAATGTCACCTTGAACTCTG | TCATTCTAGCAGGAGGAGGAC |
| CCNG1 | GCAACTGACTTGATCCGAATA | CACACCTTCTCCAATACAATCT |
| TBL1XR1 | CACCCGCTGCATTGATTTCTA | TACGGCATCTATCAGGGACAG |
| MAPK9 | AGCCAACTGTGAGGAATTATGT | GCTTGTCAGGATCAATCACTAAC |
| SERPINE1 | GCACCACAGACGCGATCTT | ACCTCTGAAAAGTCCACTTGC |
| BBC3 | TGAAGAGCAAATGAGCCAAACG | CAGAGCACAGGATTCACAGTCT |
| GTSE1 | AATAATCCGGTTCCCGAACAGC | GCCAGTAAGTGAGCTTCTTTGT |
| BAX | CCCGAGAGGTCTTTTTCCGAG | CCAGCCCATGATGGTTCTGAT |
| SESN2 | TCTTACCTGGTAGGCTCCCAC | AGCAACTTGTTGATCTCGCTG |
| RAC2 | GAAGCATCTACCCGTTCACTC | AGTTGTGGCAGCAACCATCT |
| CHEK1 | ACCTGCTTTACATTTCCACTTG | ACAGCAAACAGAGGAGGTTATT |
| JUN | ATGGTCAGGTTATACTCCTCCTC | CACATGCCACTTGATACAATCC |
| ZEB1 | CAGGCAGATGAAGCAGGATG | CAGCAGTGTCTTGTTGTTGTAG |
| PPP3CB | GGTCTTTACCGTTTGTTGGA | AGTCAGGCCCTTGAGTGTCA |
| CAMK2D | CCAGGTTCACGGACGAGTAT | GCTTCAAAAGACGGCAGATT |
| IGFBP3 | CAGAGCACAGATACCCAGAACT | GGACTCAGCACATTGAGGAAC |
| WNT5A | CAATGTCTTCCAAGTTCTTCC | GGTTATTCATACCTAGCGACC |
| PTEN | AAGACCATAACCCACCACA | ATTACACCAGTTCGTCCCT |
| DKK3 | CCGAGAAATTCACAAGATAACC | CTGGCAGGTGTACTGGAAGC |
| DKK1 | AGCGTTGTTACTGTGGAGAAG | TTGGAAGGTGTTATTGGAATGC |
| CSNK2A2 | TCGTATCGCAGAAGTTTGTC | TGGAGTTTGGGCTGTATGTT |
| SNAI2 | CAAGGACACATTAGAACTCACAC | CTACACAGCAGCCAGATTCC |
| CDKN1A | GTGACAGCGATGGGAAGG | TAGGCGGTTGAATGAGAGG |
| TP53 | ACAGCTTTGAGGTGCGTGTTT | CCCTTTCTTGCGGAGATTCTCT |
| GADD45B | CCCGCACGATGTTGATGT | CGGCCAAGTTGATGAATGT |
| CSNK2B | AGCCGAGATGCTTTATGG | GTGTCTTGATGACTTGGGTG |
| RRM2 | AAGAAACGAGGACTGATGC | CTGTCTGCCACAAACTCAA |
| CCND1 | GGTGGCAAGAGTGTGGAG | CCTGGAAGTCAACGGTAGC |
| CCND2 | CATTTCAGGCACAACGATAC | CATTTGCTGATGGCTTCTC |
| FOSL1 | ACCCTCCCTAACTCCTTTCA | CTGGAGTTGGATGTGGGATA |
| GADD45A | GAGAGCAGAAGACCGAAAGG | CAGCAGGCACAACACCAC |
| CCNB1 | CTAAGATTGGAGAGGTTGATGTC | GGTAATGTTGTAGAGTTGGTGTC |
| CSNK1E | CGCCAGAAGTATGAACGGAT | GCGGCAGAAGTTGAGGTATG |
| SKP1 | ACCCAGTTCCTCTACCAAAT | CAGCCAGAATGAGTTCAAAA |
| PORCN | TTGATGTCGATGTGGATGAC | AGCCTATGAGACGGTAGAAG |
| PPP3CC | ATTGGGAAGATGGCACGGGTCT | TTCGGGGTGGCATTCGCTCA |
| EFNB1 | GGAAATCCGCTTCACCATCA | CTCCCGGTTCTCCAGTCCCT |
| GAPDH | TGACTTCAACAGCGACACCCA | CACCCTGTTGCTGTAGCCAAA |
| SLC39A4 | CGGCGATGTTGAAAGTACG | ACAGCAGCAGCAGGACGGT |

**Supplement Table 6**

**Supplement Table 6** Antibodies used in the Western blot

| Primary antibody | Brand | Item No. | Degree of dilution |
| --- | --- | --- | --- |
| Ephrin B1 | Abcam | ab99029 | 1: 800 |
| p-Ephrin B1 | Abcam | ab30563 | 1:1000 |
| p-Ephrin B | CST | 3481 | 1:1500 |
| SLC39A4 | Thermo Fisher | PA5-69274 | 1: 500 |
| Wnt5a | Abcam | ab229200 | 1: 200 |
| p-β-catenin | CST | 4176 | 1: 1000 |
| HA Tag | Abcam | ab9110 | 1: 5000 |
| Myc Tag | Abcam | ab9106 | 1: 5000 |
| Flag Tag | Abcam | ab205606 | 1: 2000 |
| β-catenin | CST | 8480 | 1: 4000 |
| DKK1 | Abcam | ab93017 | 1: 1000 |
| DKK3 | Abcam | ab126080 | 1: 1000 |
| Snail | CST | 3879 | 1: 1000 |
| Pirh2 | Abcam | ab189247 | 1: 1000 |
| ROR2 | CST | 88639 | 1: 1000 |
| GAPDH | Abcam | ab181602 | 1: 10000 |
| p-JNK | CST | 9255 | 1: 1000 |
| JNK | CST | 9252 | 1: 1000 |
| ZEB1 | CST | 70512 | 1:1000 |
| MKK4 | CST | 9152 | 1:1000 |
| TAK1 | CST | 5206 | 1:1000 |
| Snail2（Slug） | Abcam | ab27568 | 1:500 |
| N-Cadherin | Abcam | ab207608 | 1: 1000 |
| E-Cadherin | CST | 14472 | 1: 1000 |
| FOSL1（FRA1） | Abcam | ab50426 | 1:500 |
| MMP8 | Abcam | ab81286 | 1:2000 |
| MMP9 | Abcam | ab283594 | 1: 1000 |
| ZO-1 | Abcam | ab190085 | 1:500 |
| Vimentin | Abcam | ab92547 | 1: 1000 |
| β-actin | Abcam | ab68477 | 1: 10000 |
| HA Tag | Abcam | ab9110 | 1: 5000 |
| Myc Tag | Abcam | ab9106 | 1: 5000 |
| Goat anti-Rabbit IgG（H+L）(HRP) | Thermo Pierce | 31210 | 1: 5000 |
| Goat anti-Mouse IgG（H+L）(HRP) | Thermo Pierce | 31431 | 1: 5000 |

**Supplementary Figure** **legend**

**Supplementary Figure 1** The downstream genes of ZIP4 were analyzed and verified by whole gene expression profile chip (A) Interference and overexpression efficiency of ZIP4 was detected by PCR. (B) Heat map of differentially expressed genes (DEGs) after transfection with interference lentivirus (sh-ZIP4) analyzed by Gene microarray. (C) Cluster analysis was performed on 40 DEGs (Fold Change > 2.5). (D) Metascape enrichment analysis was used to analysis DEGs showed that ZIP4 was associated with tyrosine kinase receptor signaling, cell migration regulation and EMT pathways. (E) Gene network diagram for functional enrichment analysis. (F) A total of 163 proteins were found to interact with ZIP4 using Uniprot database. They were intersected with differentially expressed genes, and 23 common genes were found, of which 17 were down-regulated and 6 were up-regulated. (G) The expression of 40 DEG (Fold Change>2.5) was verified by RT-PCR. ZMAT3, CCNG1, SERPINE1, BBC3, CDKN1A, SNAI2, CCND1, IGFBP3, RAC2, JNK, CAMK2D, BAX, GADD45A, PTEN, DKK3, PORCN, DKK1, EFNB1, FOSL1, PPP3CC, CSNK1E, SESN2, GADD45B, Wnt5A, ZEB1 were down-regulated and RRM2, RCHY1, CCNB1, TP53, GTSE1, TBL1XR1, CHEK1, CCND2, CSNK2A2, MMP7 were up-regulated. * P <0.05. (H) The protein expression of key 10 genes was verified by Western Blot. Ephrin-B1, Wnt5A and JNK were lower after ZIP4 knockout.

**Supplementary Figure** **2** The full length uncropped original western blots used in their manuscript
